# Immunological investigation of a multiepitope peptide vaccine candidate based on main proteins of SARS-CoV-2 pathogen

**DOI:** 10.1371/journal.pone.0268251

**Published:** 2022-06-09

**Authors:** Niloofar Khairkhah, Azam Bolhassani, Elnaz Agi, Ali Namvar, Arash Nikyar

**Affiliations:** 1 Department of Hepatitis and AIDS, Pasteur Institute of Iran, Tehran, Iran; 2 Iranian Comprehensive Hemophilia Care Center, Tehran, Iran; 3 Department of Molecular Medicine, School of Medicine, Qazvin University of Medical Sciences, Qazvin, Iran; Instituto Butantan, BRAZIL

## Abstract

Multiepitope vaccines could induce multiantigenic immunity against large complex pathogens with different strain variants. Herein, the *in silico*, *in vitro* and *in vivo* studies were used to design and develop a novel candidate antigenic multiepitope vaccine against SARS-CoV-2 pathogen. The designed multiepitope construct targets the spike glycoprotein (S), membrane protein (M), and nucleocapsid phosphoprotein (N) of SARS-CoV-2 (*i*.*e*., the S-N-M construct). This construct contains the cytotoxic T lymphocyte (CTL)-, helper T lymphocyte (HTL)-, and linear B lymphocyte (LBL)-inducing epitopes. The multiepitope *s-n-m* fusion gene was subcloned in prokaryotic (pET24a) and eukaryotic (pcDNA3.1) expression vectors. Its expression was evaluated in mammalian cell line using LL37 cell penetrating peptide. Moreover, the recombinant multiepitope S-N-M peptide was produced in *E*. *coli* strain. Finally, mice were immunized using homologous and heterologous regimens for evaluation of immune responses. Our data indicated that the multiepitope S-N-M peptide construct combined with Montanide 720 in homologous regimen significantly stimulated total IgG, IgG2a, IFN-γ, TNF-α, IL-15, IL-21 and IL-6, and Granzyme B secretion as compared to other groups. Moreover, the pcDNA-*s-n-m*/ LL37 nanoparticles significantly induced higher immune responses than the naked DNA in both homologous and heterologous regimens. In general, our designed multiepitope vaccine construct can be considered as a vaccine candidate in SARS-CoV-2 infection model.

## Introduction

Severe acute respiratory syndrome coronavirus 2 (SARS-CoV-2) is the causative agent of the coronavirus disease-2019 (COVID-19) [1]. This virus has five major genes with corresponding coded proteins including the orf1ab gene for orf1ab polyprotein replicase, the S gene for surface glycoprotein (known as spike protein), E gene for envelope protein, M gene for membrane protein, and N gene for nucleocapsid phosphoprotein [2]. The S glycoprotein is involved in viral pathogenesis through activation of endoplasmic reticulum (ER) stress response and thus any mutational change may lead to altered pathogenesis. The homodimeric M protein along with other viral structural proteins (such as nucleocapsid) facilitates the assembly of virus particles, and may be involved during pathogenesis. M protein possesses diverse amino acid composition, but it is structurally preserved across the various genera. N protein participates in RNA packaging, and is involved in organization of viral genome. Moreover, N protein facilitates virion assembly, and enhances virus transcription efficiency [3]. The entry of SARS-CoV-2 into the host cell is mediated by the attachment of S glycoprotein to the angiotensin-converting enzyme 2 (ACE2) receptors which are mainly expressed in type 2 alveolar cells of lungs [4, 5], thus the protective function of ACE2 is inhibited leading to vascular occlusion [6].

Since the beginning of the COVID-19 pandemic, the world has taken significant measures to cope with the disease as it caused widespread health, social and economic disruption. These include personal protection equipment, emphasizing the importance of social distancing, emergency use authorization (EUA) of remdesivir for treatment of hospitalized patients and more importantly urgent need for vaccination [7]. Major efforts are in progress to establish vaccine products for clinical usage. The authorized/approved vaccines include Comirnaty (BNT162b2), mRNA-1273, CoronaVac, COVID-19 Vaccine AstraZeneca (AZD1222), Sputnik V, BBIBP-CorV, EpiVacCorona, and Covaxin, while those in the development include Convidicea (Ad5-nCoV), JNJ-78436735 (formerly Ad26.COV2.S), INO-4800, VIR-7831, CVnCoV, and ZyCoV-D [2]. Despite the rapid production and distribution of vaccines, vaccine hesitancy, new SARS-CoV-2 variants emergence and reduced vaccine effectiveness against these emerging variants are adding further complexity [8]. So, it’s vital to understand the mechanism of current vaccines being produced as well as investigate new research for potential new vaccines to prevent another wave of the current global pandemic. Peptide-based vaccines can be designed to target different strains by the use of multiepitope approaches [9]. The understanding of epitope interaction with major histocompatibility complex (MHC) is necessary to design a vaccine being able to induce strong and long-lasting immunity against the desired pathogen. As known, multiepitope vaccines could induce multiantigenic immunity against large complex pathogens with different strain variants. However, to attain immunologic vaccine against COVID-19 pandemic, polyepitope vaccine candidate that can induce antibody from T-cell epitopes is important, as well [2]. In previous study of our team [10], we used computational approaches to identify multiepitope vaccine candidates against SARS-CoV-2 based on S, N and M proteins. Herein, we applied immunoinformatic analyses to design a multiepitope B- and T-cell candidate vaccine from three major virus proteins (S, N & M) by fusion of the immunogenic short epitopes using different linkers. Homologous and heterologous immunization regimens including DNA prime/ DNA boost, DNA prime/ Peptide boost, and Peptide prime/ Peptide boost were injected in BALB/c mice. We used LL37 peptide for delivery of the multiepitope DNA construct *in vitro* and *in vivo*. LL37 (37-amino acid; 4 kDa) is the sole member of human cathelicidin family of antimicrobial peptides, which is generated by extracellular cleavage of the C-terminal end of the human cationic antibacterial protein of 18 kDa. This cationic peptide can be found in various epithelial and immune cells including neutrophils, macrophages/monocytes, mast cells, natural killer cells and B lymphocytes [11–13]. Several studies confirmed the potential of LL37 as cell penetrating peptide (CPP) for delivery of various nucleic acid cargos [14–19]. In this study, different immunological parameters such as cytokine secretion (IFN-γ, IL-5, IL-6, IL-10, IL-15 and IL-21), antibody response (total IgG, IgG1 and IgG2a), and Granzyme B secretion (*in vitro* CTL assay) were assessed in BALB/c mice.

## Materials and methods

### Design of the multiepitope S-N-M peptide

**Fig 1:**
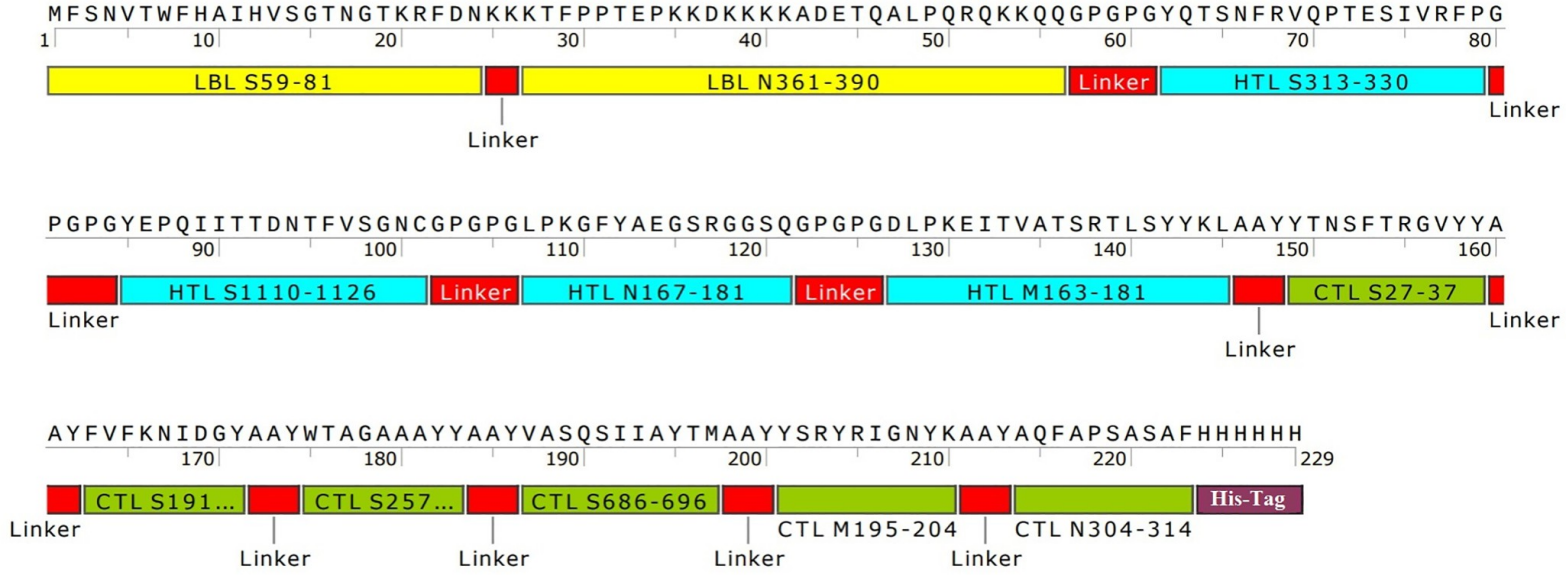
The designed multiepitope peptide construct: The schematic diagram of the construct contains LBL epitopes derived from the S and N proteins of the SARS-CoV-2 linked by KK linker, and HTL and CTL epitopes derived from the S, N and M proteins of the SARS-CoV-2 linked by GPGPG and AAY linkers, respectively. The designed construct starts with start Methionine codon and ends with His-tag. LBL: Linear B lymphocyte; HTL: Helper T-lymphocyte; CTL: Cytotoxic T-lymphocyte.

In our previous study [10], the SARS-CoV-2 sequences of S, N and M proteins were obtained from NCBI database. We intended to design a universal SARS-CoV-2 vaccine for induction of B- and T-cell immunity. Briefly, we used BepiPred tool to predict putative B-cell epitopes. As CD8^+^ and CD4^+^ T-cells play a major role in antiviral immunity, we evaluated the binding affinity to MHC class I and II molecules by NetMHCpan and NetMHCIIpan prediction tools. After using *in silico* tools such as NetCTL, tap transport/proteasomal cleavage, Pa^3^P, GalexyPepDock, I-TASSER, Ellipro and ClusPro, analysis of population coverage, and epitope conservancy, we proposed three different constructs based on linear B lymphocyte (LBL), cytotoxic T lymphocyte (CTL) and helper T lymphocyte (HTL) epitopes. Herein, to design the multiepitope peptide construct (vaccine candidate construct: COVID-19 S-N-M), the predicted B- and T-cell epitopes of S, N and M proteins with higher scores from *in silico* analyses (our previous study [10]) were fused in tandem using the KK, GPGPG and AAY proteolytic linkers. The selection of epitopes was based on immunodominant epitopes with higher MHC binding rank and population coverage (Fig 1).

### The physicochemical parameters

The physicochemical properties of designed construct including molecular weight, theoretical PI, positive and negative charge residue, solubility and stability were evaluated by ProtParam online server (https://web.expasy.org/protparam/) [20].

### Prediction of antibody-specific epitopes

IgPred module [21] (https://webs.iiitd.edu.in/raghava/igpred/index.html) was developed for predicting different types of B-cell epitopes inducing different classes of antibodies. We used this server to identify epitope tendencies for inducing IgG and IgA antibodies.

### Prediction of cytokine inducer peptides

It is important to understand that all MHC class II binders will not induce the same type of cytokines. Thus, we used IL-10 Pred [22] (http://crdd.osdd.net/raghava/IL-10pred/), IL-4 Pred (https://webs.iiitd.edu.in/raghava/il4pred/design.php) [23] and IFNepitope web server [24] (http://crdd.osdd.net/raghava/ifnepitope/index.php) to predict IL-10, IL-4 and Interferon-γ inducing peptides, respectively. We used the Support Vector Machine (SVM)-based model as prediction model in both servers. Other features such as SVM threshold left at the default value. Through using these servers, we improved insight into the future *in vivo* studies.

### 3D structure prediction

RoseTTAFOLD server [25] (https://robetta.bakerlab.org/) was used for accurately modeling the 3D structure of the designed construct. This server is in active development with the goal to provide the most accurate predictions of protein structure and function using state-of-the-art algorithms. After analysis, the models with the highest confidence score (C-score) were selected for refinement analysis.

### Refinement and validation of tertiary structure

GalaxyRefine 2 Server [26] (http://galaxy.seoklab.org/cgi-bin/submit.cgi?type=REFINE2) was used to refine predicted tertiary structures. GalaxyRefine2 performs iterative optimization with several geometric operators to increase the accuracy of the initial models. Final Refined models were analyzed by SAVES6.0 (https://saves.mbi.ucla.edu/) server to validate tertiary structures. SAVES server gives Ramachandran plot of the whole structure, determines the overall quality of tertiary structure, and calculates buried protein atoms, stereochemical quality, and atomic interaction of predicted 3D structure.

### Discontinuous B-cell epitope prediction

Prediction of discontinuous B-cell epitope needs the tertiary structure of a protein or polypeptide since the interaction between antigen epitopes and antibodies are very important. As regards, after refinement and validation analysis, the 3D structure of the designed construct was assessed by the Ellipro server [27] (https://tools.iedb.org/ellipro/help/) to find discontinuous B-cell epitopes. ElliPro web-based server uses modified Thornton’s method along with residue clustering algorithms. In this study, epitope prediction parameters (minimum score and maximum distance) were set to default values (0.5 and 6).

### Docking between vaccine construct and Toll-like receptors (TLRs)

TLRs are sensors recognizing molecular patterns of pathogens to initiate the innate immune system. It was demonstrated that TLRs 2, 3 and 4 are more susceptible to *Coronaviridae* family including SARS-CoV and MERS-CoV. Thus, PDB files of TLRs 2, 3 and 4 were obtained from Protein Data Bank (http://www.rcsb.org/) and then protein-protein docking between vaccine construct and TLRs were performed by ClusPro server [28] (https://cluspro.bu.edu/). ClusPro uses three steps algorithms containing 1) Rigid-body docking, 2) Cluster retained conformations, and 3) Refine by CHARMM minimization.

### Construct against variants of concern

The rush to eliminate the COVID-19 pandemic started as a means to administer the vaccines to protect the public from the known strain of SARS-CoV-2 that was ravaging the population. Since 2021 which most of the vaccines were developed, numerous new strains of SARS-CoV-2 have emerged. On May 2021, the World Health Organization (WHO) announces Greek letter alphabets for SARS-CoV-2 variants. At this moment, the predominant variant of SARS-CoV-2 is the delta (B.1.617.2) and omicron (B.1.1.529). Data indicate that Moderna, Pfizer-BioNtech and AstraZeneca vaccines show decreased neutralization against the delta and omicron variants of SARS-CoV-2 [29–32]. In order to investigate the fact whether our designed construct can provide immunity against delta and omicron variants, we tried to align the designed construct with their sequences. Using NCBI blast, we used the structural proteins of delta and omicron variants aligned with predicted epitope of the designed construct to identify any mutation in these regions.

### Preparation of the multiepitope DNA constructs

The nucleotide sequence of COVID-19 S-N-M was retrieved by amino acid reverse translation tool (http://www.bioinformatics.org/sms2/rev_trans.html; named as COVID-19 *s-n-m*), and the restriction enzyme sites were determined for cloning procedure (S1 Fig). The COVID-19 *s-n-m* gene was commercially synthesized by Biomatik Corporation (Cambridge, Canada) in the pUC-57 cloning vector (pUC-*s-n-m*).

### Subcloning the COVID-19 *s-n-m* gene in eukaryotic expression vector

For generation of eukaryotic expression vector harboring COVID-19 *s-n-m* gene (pcDNA-*s-n-m*), the multiepitope *s-n-m* fusion gene was subcloned from pUC-*s-n-m* into the *Bam*HI/*Hind*III cloning sites of pcDNA3.1 (-) expression vector (*In vitrogen*). The multiepitope DNA construct was confirmed by restriction enzyme digestion and sequencing. Then, the endotoxin-free plasmid (pcDNA-*s-n-m*) was generated by a Maxi-Kit DNA extraction (Qiagen). Finally, its concentration and purity was revealed by NanoDrop spectrophotometry.

### Preparation of the LL-37/ pcDNA-*s-n-m* nanoparticles

LL37 peptide (LLGDFFRKSKEKIGKEFKRIVQRIKDFLRNLVPRTES-C-amide) was synthesized by Biomatik Company (Canada). Then, pcDNA-*s-n-m* (2 μg) was incubated with LL37 in different nitrogen to phosphate (N/P) ratios (0, 0.5, 1, 2, 5, 10, 15 & 20) at room temperature for 30 minutes. The formation of LL37/ pcDNA-*s-n-m* complexes was confirmed by gel retardation assay. Then, stability of the LL37/ pcDNA-*s-n-m* complexes against nucleolytic (DNaseI) and proteolytic (10% serum) degradation was investigated at N/P ratio of 5. Next, charge of the formed nanoparticles at N/P ratios of 0 and 5 was determined using Zetasizer Nano ZS (Malvern Instruments, UK) at room temperature. Finally, scanning electron microscopy (SEM, FEI Quanta 200 SEM, PHILIPS, USA) was utilized to analyze size and morphology of the LL37/ pcDNA-*s-n-m* complex at N/P ratio of 5.

### Cytotoxicity assay

The cytotoxic effects of LL37 peptide (12.25 μg/ μl) and the LL37/ pcDNA-*s-n-m* nanoparticles (N/P ratio of 5) were evaluated on HEK-293T cells using MTT (3-[4,5-dimethylthiazol-2-yl]-2,5 diphenyl tetrazolium bromide) assay [https://www.sigmaaldrich.com].

### *In vitro* delivery of pcDNA-*s-n-m* using TurboFect and LL37 in HEK-293T cells

The HEK-293T cells (Pasteur Institute of Iran) were grown in complete DMEM (Sigma) medium supplemented with 10% fetal bovine serum (FBS, Gibco), pen/strep (100U/ml penicillin and 0.1 mg Streptomycin; Gibco) at 37°C and 5% CO_2_ atmosphere. Then, about 6 × 10^4^ cells were seeded into each well of a 24-well plate, and transfected using TurboFect transfection reagent (based on instruction manual; Thermo Scientific, Germany), and LL37 peptide (N/P ratio of 5). The untransfected HEK-293T cell line was considered as the negative control. The cells were harvested 48 h post-transfection, washed and resuspended in PBS. The expression of multiepitope peptide construct was detected by western blot analysis using a peroxidase-conjugated anti-His antibody (Abcam).

### Expression and purification of the recombinant multiepitope S-N-M peptide

At first, the multiepitope *s-n-m* fusion gene was subcloned into the *Bam*HI*/Hind*III cloning site of the pET24a (+) expression vector. Then, the recombinant pET24a (+)-*s-n-m* vector was transformed into *Escherichia coli* (*E*. *coli*) BL21 and Rosetta strains by the heat-shock method. Next, the early cultures of the transformed bacteria (*i*.*e*., single clones) were inoculated to Ty2X medium, and incubated at 37°C and 160 rpm. When the optical density (OD) of the cultures at 600 nm was raised to 0.6–0.7, the protein expression was induced by 1mM IPTG for 2, 4 and 16 h at 37°C. The expression of the multiepitope peptide construct (COVID-19 S-N-M) was analyzed by SDS-PAGE 12.5%. The solubility of COVID-19 S-N-M was determined using lysis buffer and sonication according to Qiagen protocol. The lysates were centrifuged to isolate the pellet containing inclusion bodies, and the supernatant containing soluble proteins. The COVID-19 S-N-M peptide was soluble, thus their purification was done by affinity chromatography using Ni-NTA agarose under native conditions (Immidazole 300 mM, pH = 8) as stated in the manufacturer’s instructions (Qiagen Protocol). An imidazole-SDS-Zn reverse staining approach [33] was subsequently utilized for further purification. The purified COVID-19 S-N-M peptide was detected by SDS-PAGE and western blotting using anti-His tag antibody (Abcam), dialyzed against phosphate buffered saline 1X solution (PBS 1X), quantified by NanoDrop spectrophotometry, and stored at -70°C for long-term preservation. According to LAL assay, contamination with LPS was less than 0.5 EU/mg (QCL-1000, Lonza).

### Mice immunization

Eight groups (n = 10 mice per group) of BALB/c mice aged five to seven weeks were purchased from breeding stock maintained at Pasteur Institute of Iran. The whole process was done based on approval protocols and care of laboratory animals in the Animal Experimentation Regulations of Pasteur Institute of Iran (national guideline) for scientific purposes (Ethics code: IR.PII.REC.1399.044; Approval date: 2020-09-21). All constructs were subcutaneously (*sc*) injected at the footpad. The overall immunization studies were summarized in Table 1. The recombinant COVID-19 S-N-M (5 μg) peptide was mixed with Montanide 720 adjuvant (the peptide: adjuvant ratio is 30: 70 *v/v*). The pcDNA-*s-n-m* was used alone (50 μg) or along with LL37 peptide (5 μg DNA complexed with LL37 at N/P ratio of 5).

**Table 1:** Mice immunization program.

Group	Modality	First injection (Prime: Day 0)	Second injection (Booster 1: Day 14)	Third injection (Booster 2: Day 28)
**G1**	DNA/DNA/DNA	pcDNA-*s-n-m* + LL37	pcDNA-*s-n-m* + LL37	pcDNA-*s-n-m* + LL37
**G2**	DNA/Peptide/Peptide	pcDNA-*s-n-m* + LL37	COVID-19 S-N-M + Montanide 720	COVID-19 S-N-M + Montanide 720
**G3**	Peptide/Peptide/Peptide	COVID-19 S-N-M + Montanide 720	COVID-19 S-N-M + Montanide 720	COVID-19 S-N-M + Montanide 720
**G4**	DNA/DNA/DNA	pcDNA-*s-n-m*	pcDNA-*s-n-m*	pcDNA-*s-n-m*
**G5**	DNA/Peptide/Peptide	pcDNA-*s-n-m*	COVID-19 S-N-M + Montanide 720	COVID-19 S-N-M + Montanide 720
**G6**	Control	PBS	PBS	PBS
**G7**	Control	pcDNA3.1 (empty vector)	pcDNA3.1 (empty vector)	pcDNA3.1 (empty vector)
**G8**	Control	LL37	LL37	LL37

### Antibody assay

The blood samples from mice were collected from retro-orbital after anesthesia with ketamine/ xylazine three weeks after the last administration and also three months after. Next, the levels of goat anti-mouse total IgG, IgG1, IgG2a antibodies (1:10,000 *v/v*, Sigma) were measured in the pooled sera (1:50 *v/v*) of each group by indirect enzyme-linked immunosorbent assay (ELISA). The coated antigen was the recombinant COVID-19 S-N-M peptide (5μg/ ml).

### Cytokine secretion

Five mice from each group (n = 5 per group) were sacrificed after anesthesia with ketamine and xylazine three weeks after the last administration and also three months after, individually. After removing spleens, the pooled splenocytes of five mice (in both times) without red blood cells (2 × 10^6^ cells /ml) were seeded in 48-well plates (Greiner) exposed to the recombinant COVID-19 S-N-M peptide (5μg/ ml), RPMI 5% (negative control), and 5μg/ ml of concanavalin A (ConA, positive control) in complete RPMI medium for 72 h. The supernatants were used to assess IFN-γ, IL-5, IL-10, IL-6, TNF-α (Mabtech Swedish Biotech *Co*.), IL-15 and IL-21 cytokines (Abcam, USA) using the sandwich-based ELISA method according to the manufacturer’s instructions. The results were shown as mean ± SD for each group.

### Granzyme B assay

The SP2.0 target cells (T: 3 × 10^4^ cells/well) were incubated with the recombinant COVID-19 S-N-M peptide (~10 μg/ ml) for 24 h. Then, the effector cells (E: the red blood cell-depleted pooled splenocytes) were added to the target cells at E: T ratio of 100:1, and incubated for six hours. Finally, the concentration of Granzyme B was assessed in the supernatants using ELISA (eBioscience kit).

### Statistical analysis

Statistical analysis was done by Prism software (GraphPad) using one-way ANOVA and student’s *t*-test. The *p-value* < 0.05 was statistically considered significant. The experiments were independently performed twice.

## Results

### Construction of vaccine candidate

According to mentioned parameters in our previous study [10] including binding affinity between peptide and MHCs, epitope identification scores for T-cells, proteasomal cleavage and Tap transport scores, conservancy degrees and population coverage, one construct was designed by top-ranked epitopes. The multiepitope peptide construct included LBL epitopes (S^59-81^, N^361-390^) linked by KK linker [34, 35], HTL epitopes (S^313-330^, S^1110-1126^, N^167-181^, M^163-181^) linked by GPGPG linker [35] and CTL epitopes (S^27-37^, S^191-199^, S^257-265^, S^686-696^, M^195-204^, N^304-314^) linked by AAY linker [35, 36] (Fig 1). To enhance expression, folding and stabilization, linkers are implemented as an indispensable element in the development of vaccine protein. The KK, GPGPG and AAY linkers play vital roles in producing and extended conformation (flexibility) and protein folding and have the capacity to improve sequence flexibility without affecting the function of proteins that they attached to [37, 38].

### The physicochemical parameters

The designed multiepitope construct was analyzed by ProtParam server. The molecular weight (MW) of the construct was 25.26 kDa with a theoretical isoelectric point (PI) of 9.67. The construct was soluble (GRAVY index: -0.644) and stable (instability index: 35.39). Also, the positive and negative charges of the peptide were predicted as 26 (Arg+Lys) and 12 (Asp+Glu), respectively.

### Prediction of antibody-specific and cytokine inducer peptides

All selected epitopes included in the designed construct were analyzed for putative antibody-specific and cytokine inducers. Table 2 shows IFN-γ, IL-10, IL-4, IgG and IgA prediction. All selected epitopes were negative for allergenicity.

**Table 2:** Prediction of antibody-specific and cytokine inducer epitopes.

Epitopes	Sequence	IFN	IL10	IL4	IgG	IgA	Allergenicity
S^59-81^	FSNVTWFHAIHVSGTNGTKRFDN	P	P	N	P	N	N
N^361-390^	KTFPPTEPKKDKKKKADETQALPQRQKKQQ	P	N	N	P	N	N
S^313-330^	YQTSNFRVQPTESIVRFP	P	P	P	N	N	N
S^1110-1126^	YEPQIITTDNTFVSGNC	P	N	P	N	N	N
N^167-181^	LPKGFYAEGSRGGSQ	P	N	N	N	N	N
M^163-181^	DLPKEITVATSRTLSYYKL	P	N	P	N	N	N
S^28-38^	YTNSFTRGVYY	P	P	N	N	N	N
S^192-200^	FVFKNIDGY	N	N	P	N	N	N
S^258-266^	WTAGAAAYY	P	N	N	N	N	N
S^687-697^	VASQSIIAYTM	P	N	N	N	N	N
M^196-205^	YSRYRIGNYK	N	N	P	N	N	N
N^305-314^	AQFAPSASAF	P	N	P	N	N	N

* P for positive prediction and N for negative prediction

### 3D structure prediction

The designed structure was analyzed by RoseTTaFOLD server. The assurance of the model was calculated by confidence score which corresponds to the agreement in structure between partially threaded models from the top alignment of each independent alignment method. The confidence scores vary between 0.0–1.0 (the higher value of the confidence score is the better quality of prediction). The confidence of the designed structure was 0.65 showing a good prediction.

### Refinement and validation of 3D structure

**Fig 2:**
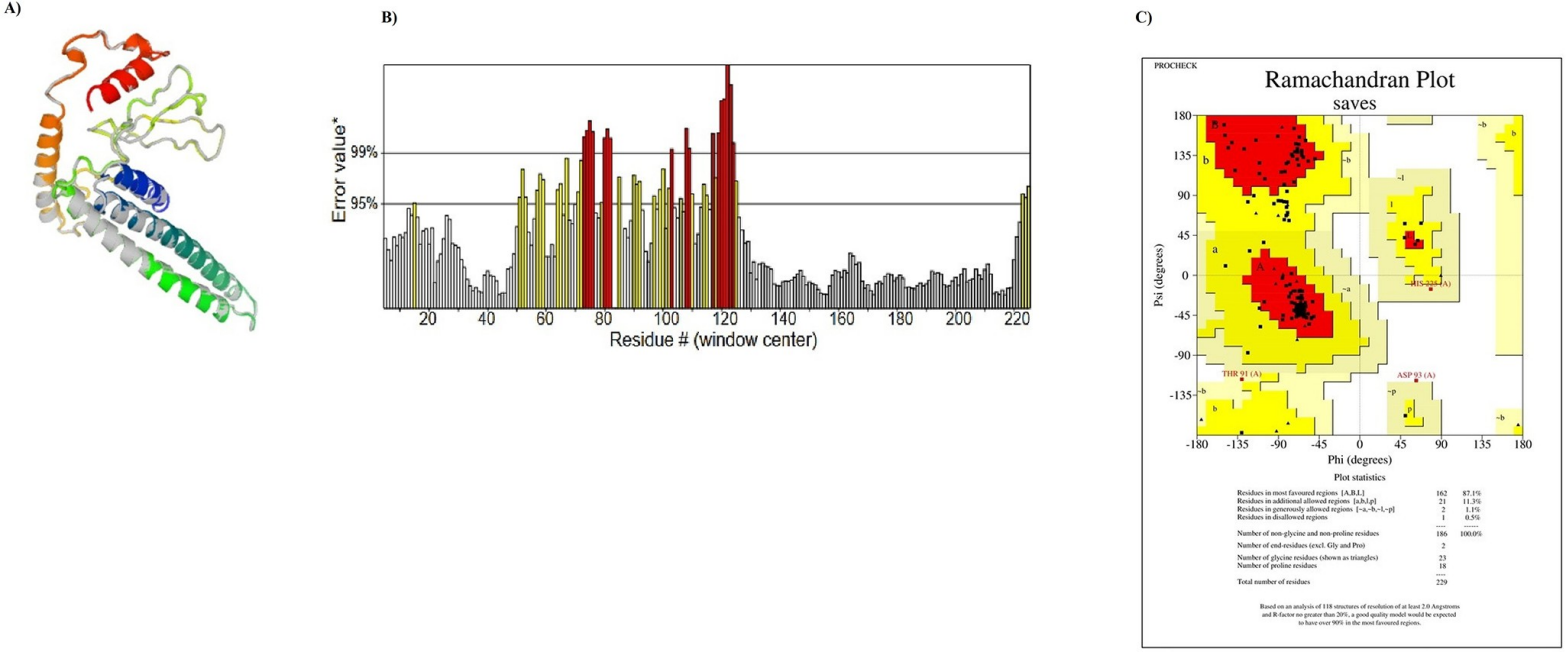
Refined characteristics of the designed construct: A) Refined 3D prediction of the designed construct: The C-terminal and the N-terminal of protein were shown as red and blue color, respectively; B) Overall quality of refinement: The red and yellow bars show error rate in predicted 3D structure. The yellow bars are errors that need to be rejected at a confidence level greater than 95%. The red bars are errors that need to be rejected at a confidence level greater than 99%; C) Ramachandran plot.

After prediction of tertiary structure, the top model of the designed construct was submitted to GalaxyRefine 2 server. Then, the refined model was submitted to SAVES6.0 server for validation. The data indicated that the quality of 3D structure was improved after the refinement process. Fig 2 shows refined characteristics including secondary structure, overall quality and Ramachandran plot. Most of the residues (99.5%) were found in favored and allowed regions.

### Prediction of linear and discontinuous B-cell epitopes

Discontinuous epitopes need 3D structural information in contrast with linear antibody epitopes which can be predicted through sequence-based algorithms. In order to predict the potential linear and discontinuous B-cell epitopes, the selected refined models were analyzed by Ellipro server. Ellipro server identified 6 linear and 5 discontinuous B-cell epitopes for the designed construct. Table 3 and S1 Table indicate residues, number of residues, and the 3D structure of putative B-cell epitopes in the designed construct.

**Table 3:** Prediction of discontinue B-cell epitopes in the designed S-N-M multiepitope peptide.

Residues	Number of Residues	Score
A:Y148, A:N151, A:S152, A:T154, A:R155, A:G156, A:V157, A:Y158, A:Y159, A:A160, A:A161, A:Y162, A:F163, A:V164, A:F165, A:K166, A:D169, A:G170, A:Y171, A:A172, A:A173, A:Y174, A:W175, A:T176, A:A177, A:G178, A:A179, A:A180, A:A181, A:Y182, A:Y183	31	0.792
A:A42, A:D43, A:E44, A:T45, A:Q46, A:A47, A:L48, A:P49, A:Q50, A:R51, A:Q52, A:K53, A:Q55, A:Q56, A:G57, A:P58, A:G59, A:G61, A:Y62, A:Q63, A:T64, A:S65	22	0.744
A:W7, A:A10, A:I11, A:H12, A:V13, A:S14, A:G15, A:T16, A:N17, A:G18, A:T19, A:K20, A:R21, A:F22, A:D23, A:N24, A:K25, A:K27, A:T28, A:F29, A:P30, A:P31, A:T32, A:K35, A:K36, A:R77, A:F78, A:P79, A:G80, A:P81, A:G82, A:P83, A:G84, A:Y85, A:G99, A:N100, A:C101, A:G102, A:P103, A:G104, A:P105, A:G106, A:L107, A:P108, A:K109, A:G110, A:F111, A:Y112, A:A113	49	0.665
A:F2, A:S3, A:T6	3	0.587
A:H225, A:H227, A:H228, A:H229	4	0.505

* ElliPro’s top predictions are those with the highest scores.

### Docking between the multiepitope construct and toll-like receptors (TLRs)

**Fig 3:**
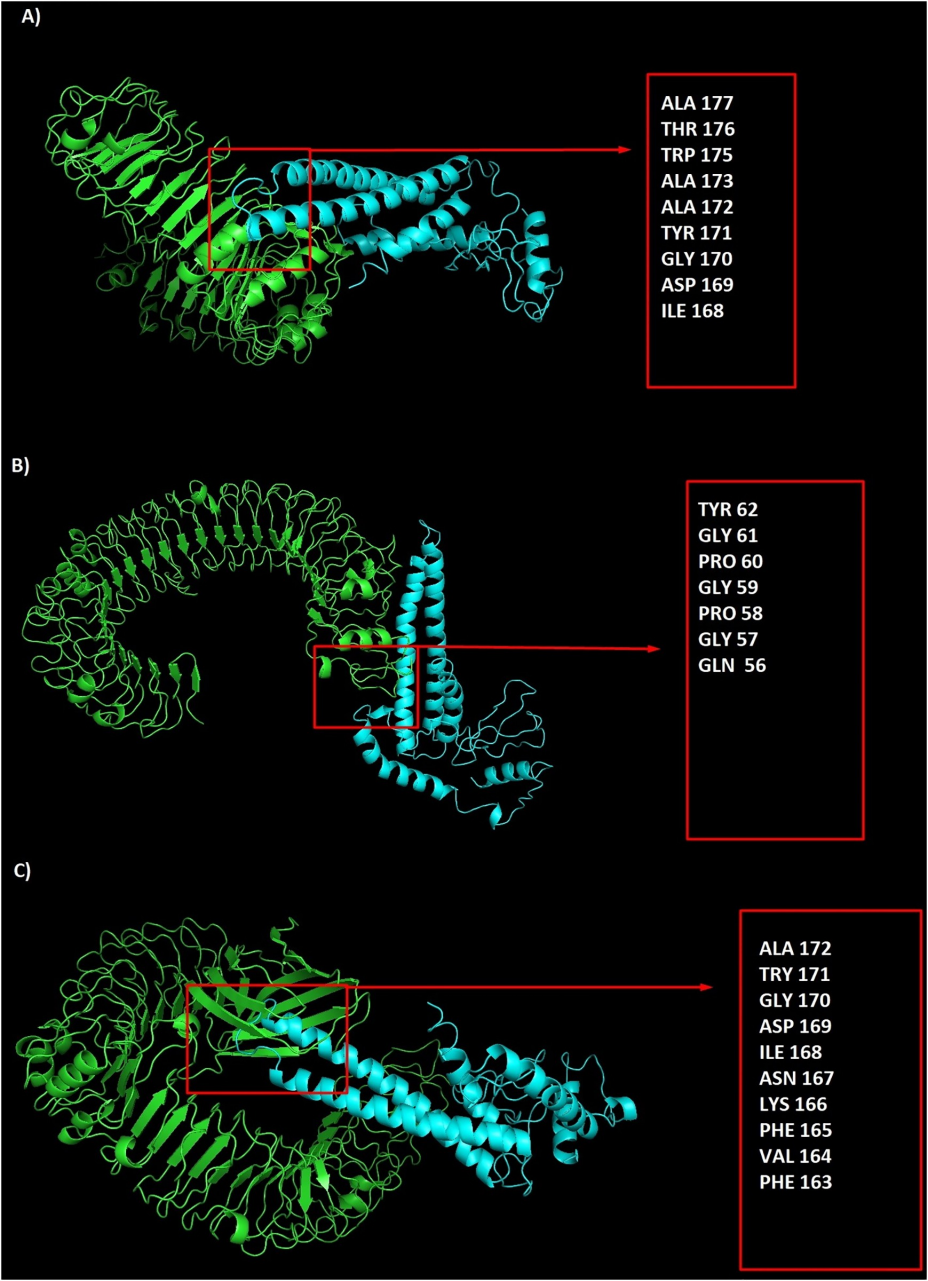
The peptide-protein docking between the designed construct and TLRs 2, 3 and 4: A) designed construct-TLR2 complex with participated residues in interaction, B) designed construct-TLR3 complex with participated residues in interaction, C) designed construct-TLR4 complex with participated residues in interaction.

The peptide-protein docking between the designed construct and TLRs 2, 3, 4 was performed by ClusPro server. The lowest energy level was estimated -1262.6, -1131.8 and -941.7 for the designed construct-TLR2 complex, construct-TLR4 complex and construct-TLR3 complex, respectively. Strong interactions between the designed construct and TLRs 2, 3 and 4 support the hypothesis of SARS-CoV-2 susceptibility to TLRs like other *Coronaviridae* families (Fig 3).

### Construct against variant of concern

**Fig 4:**
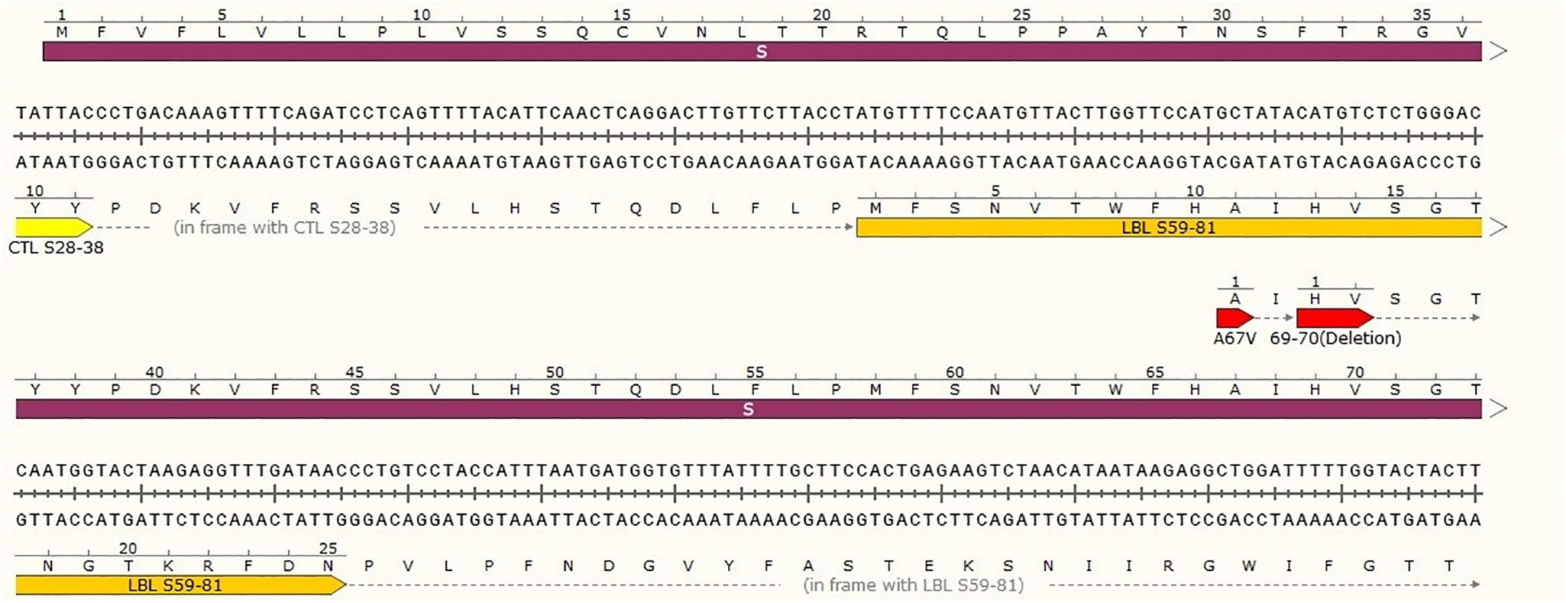
Mutation of SARS-CoV-2 delta and omicron strains in S protein of the virus. Of the twelve selected epitopes, only LBL S^59-81^ epitope contained mutation. The mutation was shown in yellow containing a substitution and two deletions.

In order to investigate the fact whether our designed construct can provide immunity against delta and omicron variants, we tried to align the designed construct with their sequences. Of the twelve predicted and selected epitopes in different area of structural proteins of SARS-CoV-2 delta and omicron strains, only B-cell S^59-81^ epitope in our designed construct contained mutations (A67V, 69del and 70 del) (Fig 4). In this case, these mutations consist of a structural and/or charge mutation which may impact antibody binding.

### Confirmation of DNA constructs

The endotoxin-free plasmid (pcDNA-*s-n-m*) and the recombinant pET24a-*s-n-m* were confirmed by enzyme digestion as a clear band of ~708 bp on agarose gel (S2 Fig). Their accuracy was confirmed by sequencing, as well. The concentration of endotoxin-free DNA was about 2084 ng/μl.

### Formation of the LL37/ pcDNA-*s-n-m* nanoparticles and their characteristics

**Fig 5:**
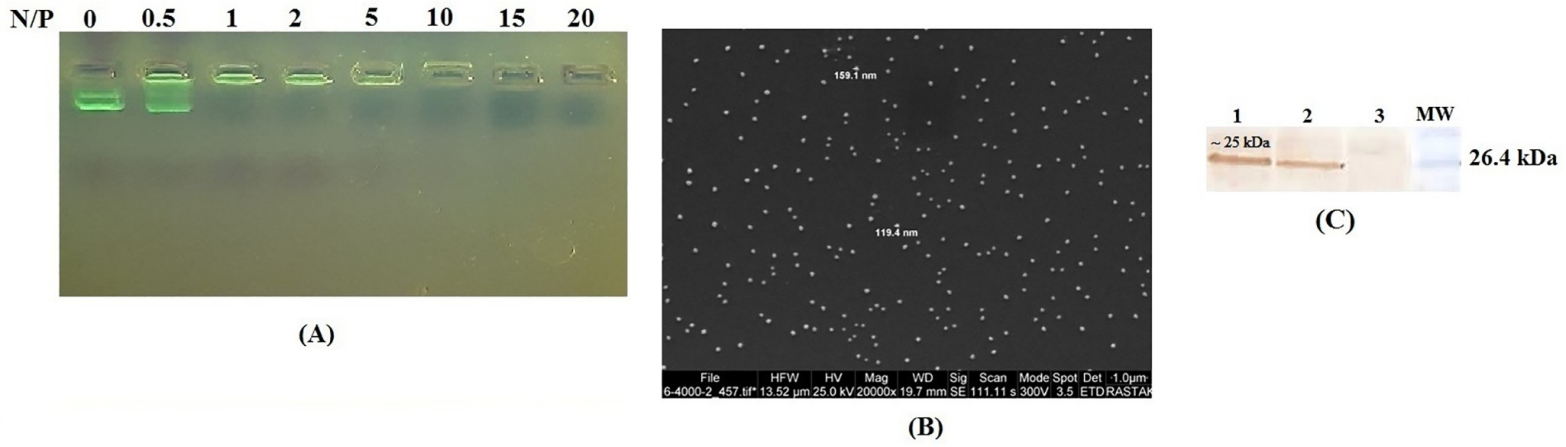
Formation of the LL37/ pcDNA-*s-n-m* nanoparticles: **A)** Gel retardation assay at N/P ratios of 0, 0.5, 1, 2, 5, 10, 15 & 20 on 1% agarose gel; **B)** SEM images of the LL37/ pcDNA-*s-n-m* nanoparticles. Images were taken under 2×10^4^ magnification; **C)** Transfection efficiency of LL37 peptide and TurboFect for delivery of pcDNA-*s-n-m* into HEK-293T cells using western blot analysis. MW: molecular weight (14.4–97.4 kDa, Fermentas); Lane 1: Cells transfected with the pcDNA-*s-n-m*/LL-37 nanoparticles; Lane 2: Cells transfected with the pcDNA-*s-n-m*/TurboFect complexes; Lane 3: Untransfected HEK-293T cells.

The LL37/ pcDNA-*s-n-m* complex was formed by co-incubation of pcDNA-*s-n-m* (2 μg) with LL37 at different N/P ratios for 30 min. As shown in Fig 5A, the LL37 peptide inhibited DNA migration in 1% agarose gel at N/P ratio of 1 and above, indicating successful formation of the LL37/DNA complexes. To evaluate the stability of the formed complexes against nucleolytic and proteolytic degradation, the formed LL37/ pcDNA-*s-n-m* complexes at a certain N/P ratio of 5 were treated with DNaseI and 10% FBS, respectively. The results showed that the LL37/ pcDNA-*s-n-m* complex was intact and had good stability against DNaseI and 10% serum compared to the degraded pcDNA-*s-n-m*. To investigate the impact of LL37 peptide on the zeta potential of the recombinant pcDNA-*s-n-m* construct, the LL37/ pcDNA-*s-n-m* complex were prepared in N/P ratios of 0 & 5, and analyzed by a Zetasizer system. The results indicated that the N/P ratio of 5 led to the positive surface charge of the nanoparticles (-16.7 for N/P ratio of 0; 14.21 for N/P ratio of 5). Based on the results of SEM, spherical LL37 (diameter: 50–96 nm) formed spherical nanoparticles (diameter of ~ 120–160 nm) when complexed with pcDNA-*s-n-m* at N/P ratio of 5 (Fig 5B).

### Cytotoxicity of the LL37/ pcDNA-*s-n-m* nanoparticles

MTT assay was performed to investigate the cytotoxic effects of LL37 peptide, pcDNA*-s-n-m* and the LL37*/* pcDNA*-s-n-m* nanoparticles on HEK-293T cells after 48 hours. Based on the results, the percentage of cell viability was ~ 30% ± 3.7, ~96% ± 1.4 and ~92% ± 1.0 for LL37 peptide, pcDNA*-s-n-m* and the LL37*/* pcDNA*-s-n-m* nanoparticles as compared to untransfected cells (~ 98% ± 1.5), respectively. Thus, LL37 significantly reduced cell viability at concentration used in N/P ratio of 5. In contrast, the LL37*/* pcDNA*-s-n-m* nanoparticles had no significant cytotoxic effect on HEK-293T cells at N/P ratio of 5.

### Transfection of HEK-293T cells with the LL37/ pcDNA-s-n-m nanoparticles

Based on the results of gel retardation assay, zeta potential analysis and MTT assay, the LL37/ pcDNA-*s-n-m* nanoparticles at N/P ratio of 5 was used for transfection of HEK-293T cells. Western blot analysis was performed to confirm the expression of the multiepitope COVID-19 S-N-M peptide in the transfected cells. The COVID-19 S-N-M peptide was detected as a clear band of *~*25 kDa in the cells treated with the LL37/ pcDNA-*s-n-m* nanoparticles, and the pcDNA-*s-n-m*/ TurboFect complex. No clear band was found in untreated HEK-293T cells (Fig 5C).

### Generation of the recombinant COVID-19 S-N-M peptide

**Fig 6:**
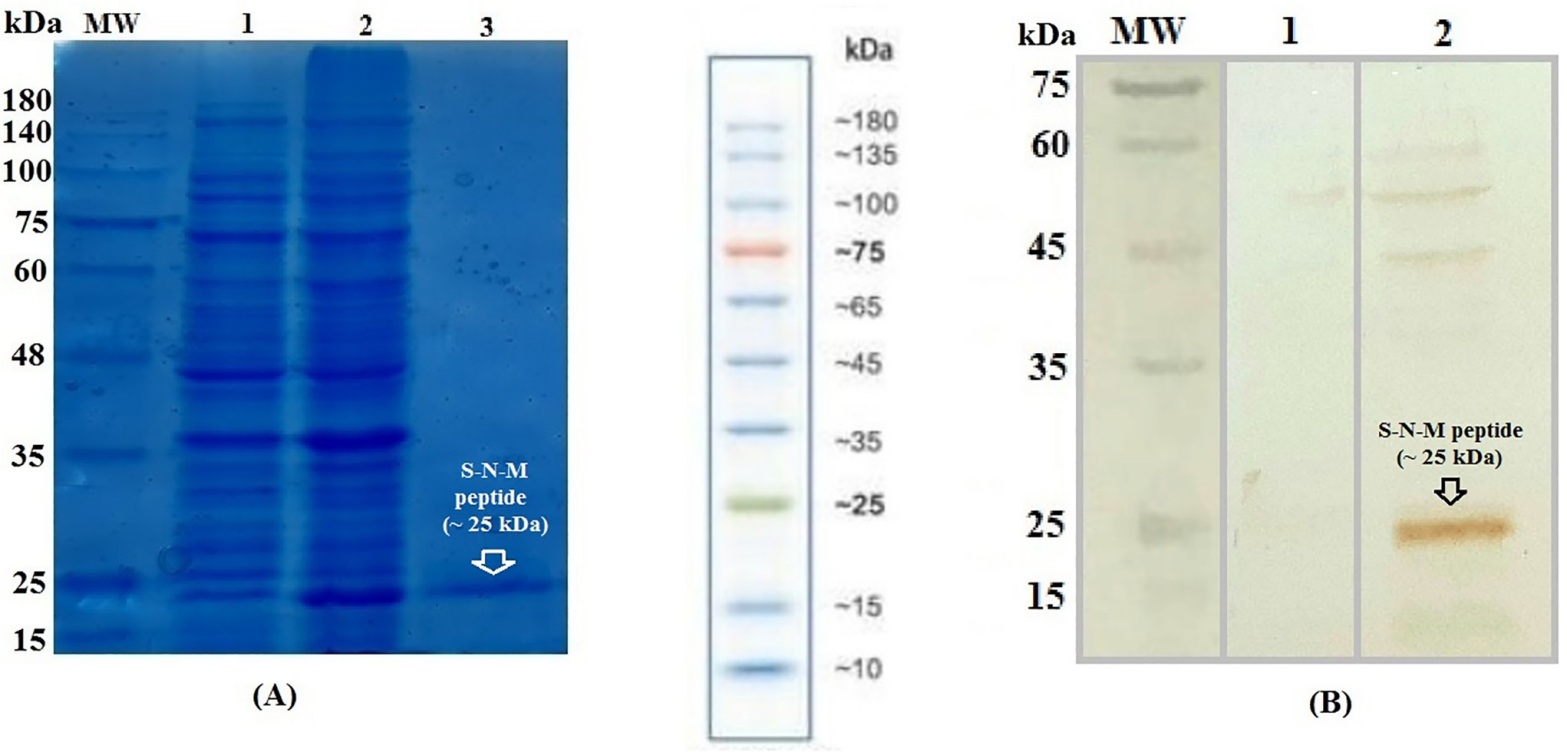
A) Generation of the recombinant S-N-M peptide in Rosetta strain: Lane 1: before induction; Lane 2: 16 hours after IPTG induction; Lane 3: Purified peptide; B) Confirmation of the expressed S-N-M multiepitope peptide using western blotting: Lane 1: before induction; Lane 2: 16 hours after induction; MW: Molecular weight marker (prestained protein ladder, 10–180 kDa, Fermentas).

The expression of COVID-19 S-N-M peptide was evaluated in Rosetta and BL21 strains using IPTG inducer. The results revealed that the expression of multiepitope peptide was performed only in Rosetta strain at 37°C, and 16 h after induction. Its expression was confirmed as a clear band of ~25 kDa in SDS-PAGE and western blotting, respectively (Fig 6). The solubility test showed that the recombinant COVID-19 S-N-M was mainly observed in the supernatant. Thus, the COVID-19 S-N-M peptide fused to 6X-His tag was effectively purified by affinity chromatography under Native conditions. Subsequently, reverse staining method was performed to remove non-specific bands and further purity. The purified COVID-19 S-N-M peptide was observed as a clear band of ~25 kDa in SDS-PAGE (Fig 6A). The concentration of purified peptide was about 0.588 mg/ ml for 100 ml of bacterial culture.

### Antibody secretion

**Fig 7:**
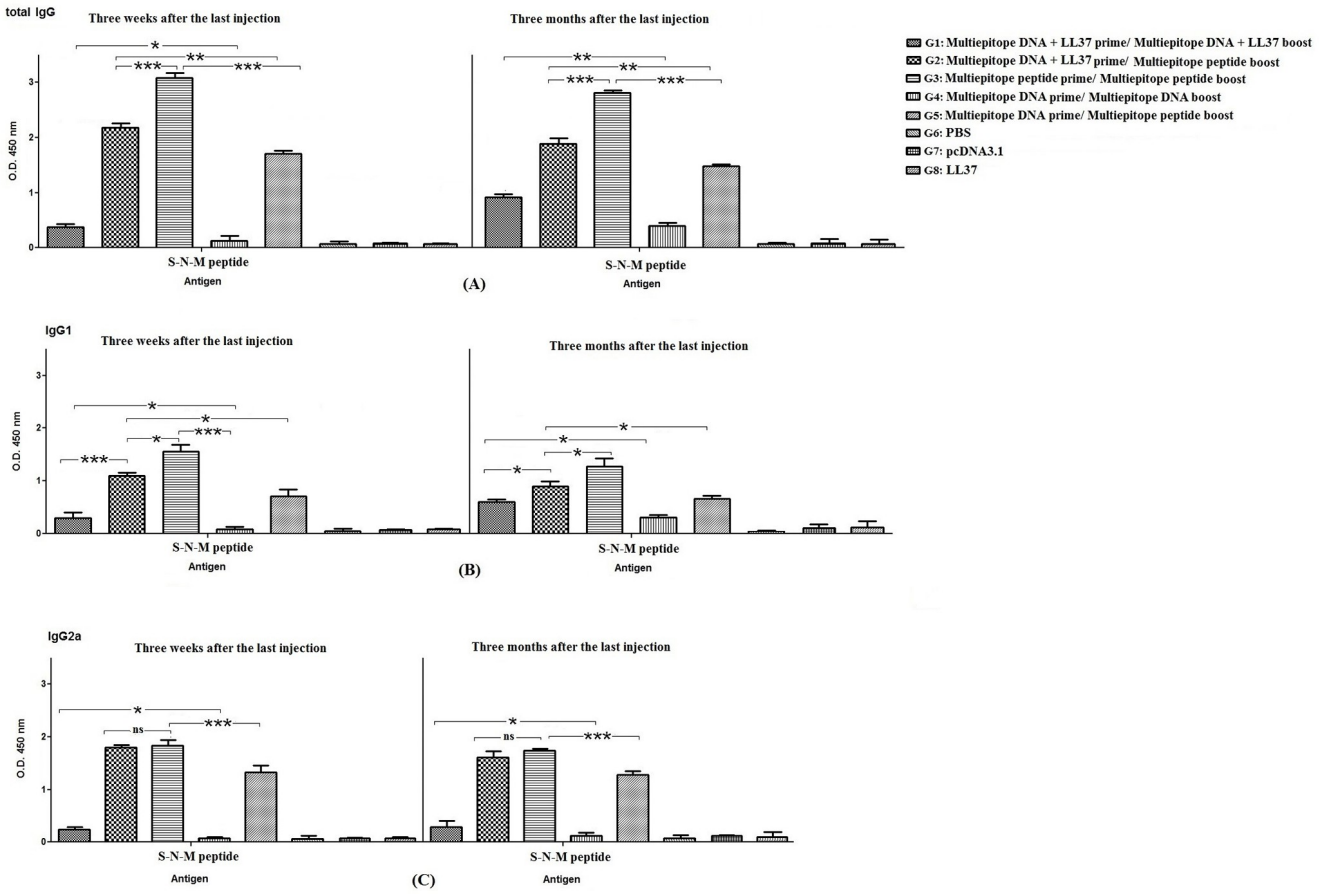
The levels of total IgG (A), IgG1 (B), and IgG2a (C) antibodies against S-N-M antigen at 3 weeks and 3 months after the last injection: Data were presented as the mean absorbance at 450 nm ± SD from two independent experiments. Significant differences were shown by * *p* < 0.05, ** *p* < 0.01, *** *p* < 0.001, and non-significant difference was shown by ns (*p* > 0.05).

The levels of total IgG and IgG1 in mice immunized with the homologous S-N-M multiepitope peptide regimen (G3) were significantly higher than other groups (*p* < 0.05 or *p* < 0.001; Fig 7). In contrast, the level of IgG2a was not considerably different in mice immunized with the homologous S-N-M multiepitope peptide regimen (G3), and mice immunized with the heterologous S-N-M multiepitope peptide regimen (DNA + LL37 prime/ Peptide + Montanide boost: G2; *p* > 0.05). It was interesting that the levels of total IgG, IgG1 and IgG2a were higher in mice immunized with DNA + LL37 prime/ Peptide + Montanide boost (G2) than mice immunized with DNA prime/ Peptide + Montanide boost (G5; *p* < 0.001). However, the mean ratio of IgG2a to IgG1 was significantly high in these groups (G2, G3 and G5) indicating direction of immune responses toward Th1 response. In addition, the LL37/ DNA nanoparticles (G1 and G2) showed higher efficiency than the naked DNA (G4 and G5) in increasing total IgG, IgG1 and IgG2a secretion (*p* < 0.05). Other data indicated that the immune responses were almost stable at 3 months after the last injection for all groups. Specially, in groups immunized with the LL37/ DNA nanoparticles (G1) and the naked DNA (G4), the levels of IgG1 and total IgG were significantly enhanced at 3 months as compared to at 3 weeks after the last injection indicating slower induction of humoral responses by the homologous DNA regimens (Fig 7).

### Cytokine secretion

**Fig 8:**
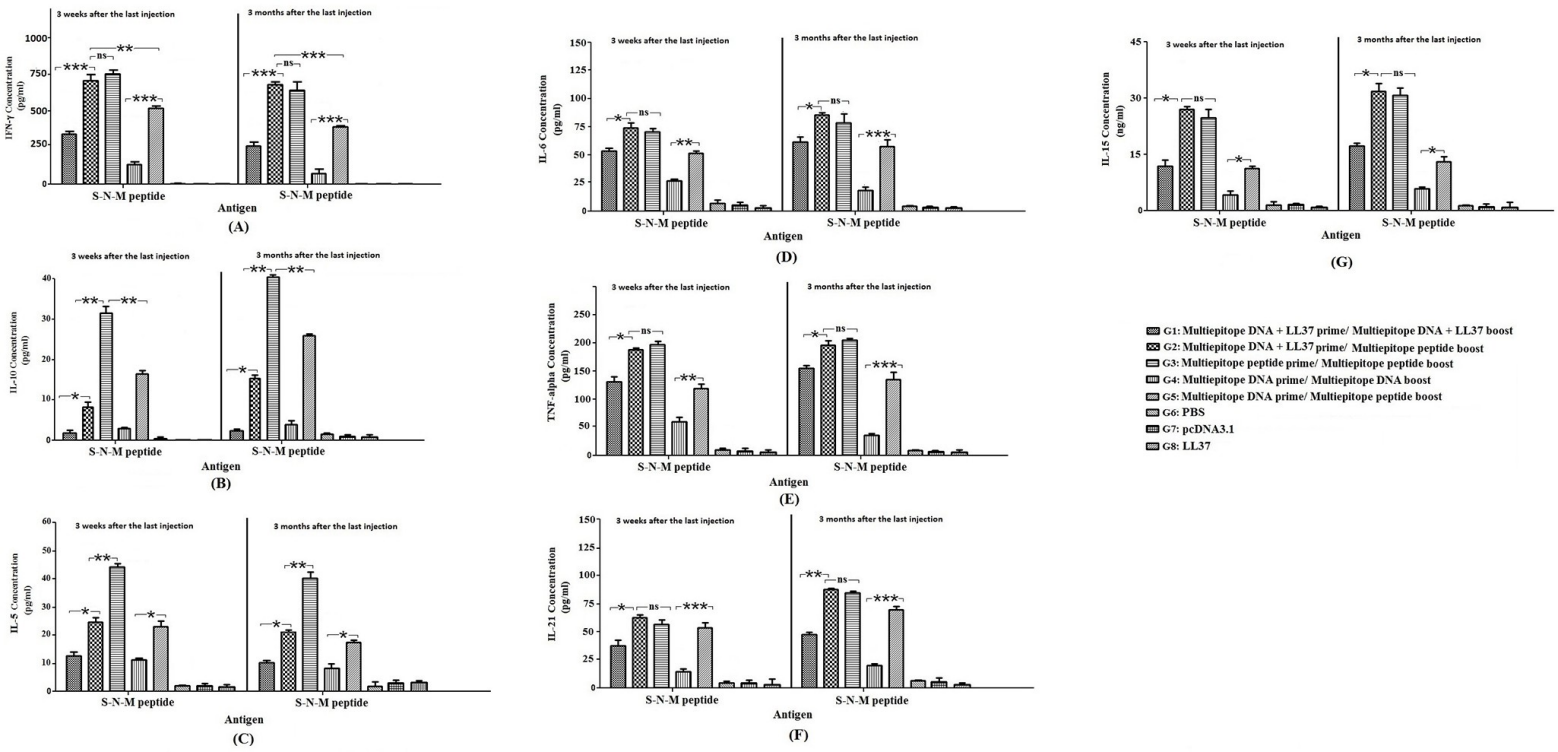
The secretion levels of IFN-γ (A), IL-10 (B), and IL-5 (C), IL-6 (D), TNF-α (E), IL-21 (F), and IL-15 (G) cytokines in splenocytes of mice immunized with S-N-M antigen in various formulations. The levels of cytokines were determined in the supernatant using sandwich-based ELISA as the mean absorbance at 450 nm ± SD from two independent experiments. Significant differences were shown by * *p* < 0.05, ** *p* < 0.01, *** *p* < 0.001, and non-significant difference was shown by ns (*p* > 0.05).

The highest level of IFN-γ cytokine was observed in the heterologous multiepitope DNA prime/multiepitope peptide boost regimen (G2) and the homologous multiepitope peptide prime/multiepitope peptide boost (G3) compared to other groups (*p* < 0.001; Fig 8). No significant difference was observed in the levels of IFN-γ, TNF-α, IL-15, IL-21, and IL-6 between groups 2 and 3 (G2 & G3; *p* > 0.05). In contrast, the levels of IL-5 and IL-10 in group 3 (G3) was higher than those in group 2 (G2; *p* < 0.05). Moreover, LL37 could significantly increase the efficiency of DNA construct (G1 & G2) as compared to the naked DNA (G4 & G5) in the release of IFN-γ, TNF-α, IL-15, IL-21, and IL-6 cytokines (*p* < 0.05; Fig 8).

### Secretion of Granzyme B and lymphocyte proliferation

**Fig 9:**
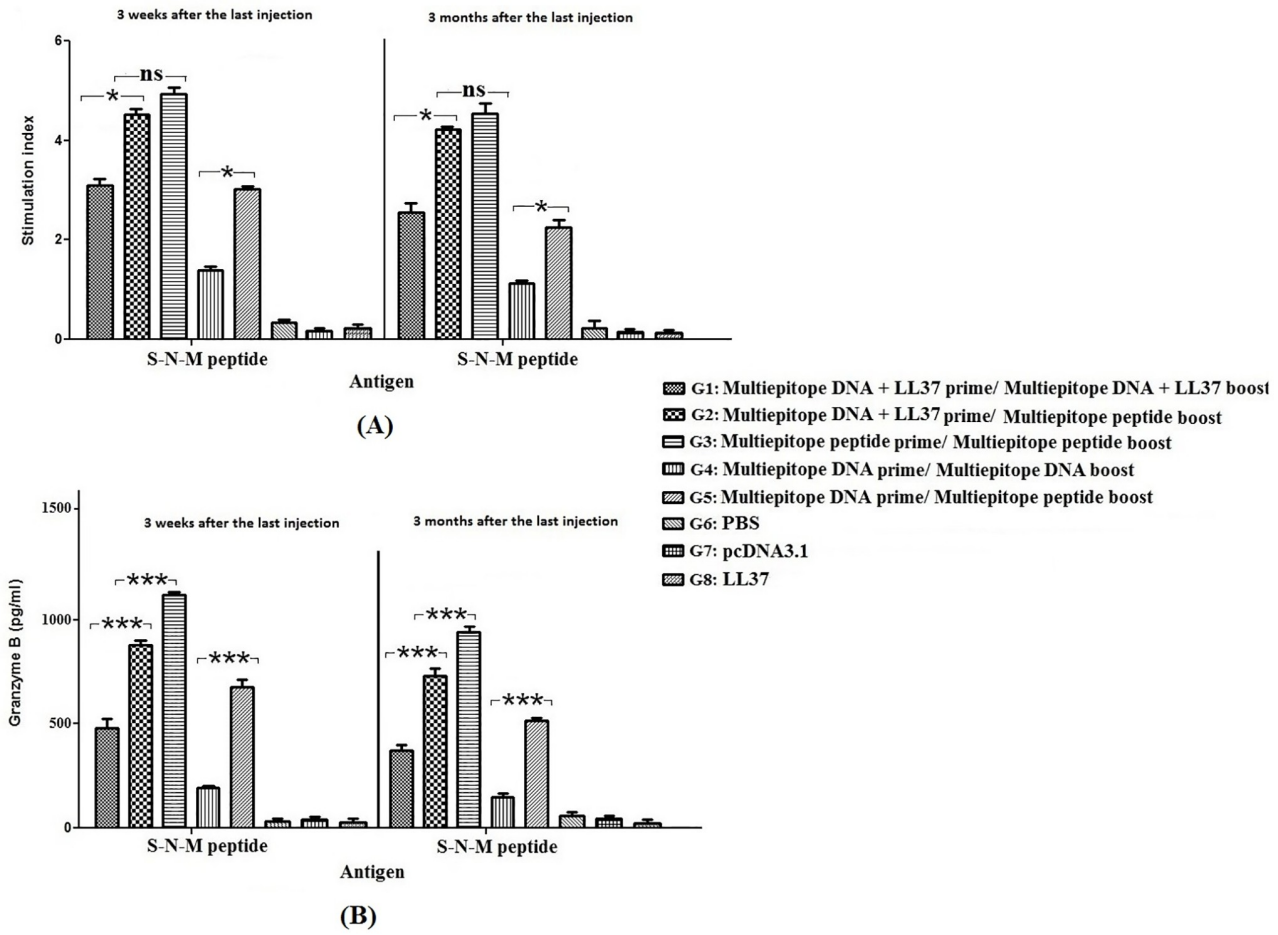
The lymphocyte proliferation (A) and Granzyme B concentration (B) in immunized groups with S-N-M antigen in various formulations. The Granzyme B concentration was determined as the mean absorbance at 450 nm ± SD from three independent experiments. Significant differences were shown by * *p* < 0.05, ** *p* < 0.01, *** *p* < 0.001, and non-significant difference was shown by ns (*p* > 0.05).

The highest secretion of Granzyme B and the lymphocyte proliferation was observed in group immunized with the homologous multiepitope peptide prime/multiepitope peptide boost (G3). Moreover, LL37 peptide could significantly increase the efficiency of DNA construct (G1 & G2) as compared to the naked DNA **(**G4 & G5; Fig 9).

## Discussion

There are still many questions posed by the SARS-CoV-2 vaccine effort that need to be addressed in the context of the pandemic. The emergence of the new strains has been shown to affect the current therapeutics, indicating the current vaccines can become inefficient. In this sense, being able to achieve the production of a vaccine that can induce a potent and long term immune response against different variants of SARS-CoV-2 even targeting the entire *coronavirus* family is not far-fetched. Moreover, development of a therapeutic vaccine is necessary for inducing strong immune responses in infected subjects. Peptide-based vaccines can be designed to target different strains by the use of multiepitope approaches. The understanding of epitope interaction with major histocompatibility complex (MHC) is necessary to design a vaccine being able to induce strong and long-lasting immunity against the desired pathogen. In the previous study of our team [10], we used computational approaches to identify multiepitope vaccine candidates against SARS-CoV-2 based on S, N and M proteins. We identified epitopes corresponding to B- and T-cells to design constructs being able to elicit both humoral and cellular immunity. We chose immunodominant epitopes with higher MHC binding rank and population coverage of each LBL, CTL and HTL construct of our previous study [10] as one construct including B-cell epitopes (S^59-81^, N^361-390^), helper T-cell epitopes (S^313-330^, S^1110-1126^, N^167-181^, M^163-181^) and cytotoxic T-cell epitopes (S^27-37^, S^191-199^, S^257-265^, S^686-696^, M^195-204^, N^304-314^). The tendency to induce IFN-γ, IL-10 and IL-4 cytokines and IgG and IgA antibodies were predicted, as well. Another prominent obstacle in vaccine development is the probability of allergenicity since many vaccines stimulate the immune system into an allergenic reaction. In this study, all of the selected epitopes were analyzed as non-allergen. The vaccine construction was completed after joining the LBL, HTL and CTL epitopes with KK, GPGPG and AAY linkers, and a methionine start codon at the initiation site. Linkers (AYY, KK, and GPGPG) play vital roles in producing an extended conformation (flexibility), protein folding, and separation of functional domains, and therefore, make the protein structure more stable. We used these linkers which have been reported in many studies [34–39]. The physicochemical properties of the designed construct including molecular weight, theoretical PI, positive and negative charge residues, solubility and stability were also evaluated. The molecular weight of the designed construct was estimated as 25.26 kDa with PI 9.67. The designed construct showed high solubility and stability for initiation of an immunogenic reaction.

In the case of 3D modeling, we used RoseTTAFOLD server to predict the tertiary protein structure. The assurance of the model was calculated by confidence score varying between 0.0–1.0 (the higher value of the confidence score is the better quality of prediction). The confidence of the designed structure was 0.65 showing a good prediction. Also, the quality of the predicted construct was improved by refinement leading to a higher quality of final models. At last, we used Ellipro server to predict linear and discontinuous B-cell epitopes. Ellipro server identified 6 linear and 5 discontinuous B-cell epitopes for the designed construct indicating the ability of the designed construct for robust induction of humoral response. Also, peptide-protein docking between vaccine construct and TLRs 2, 3 and 4 were performed by ClusPro server, and all data showed strong interactions between the designed construct and TLRs 2, 3 and 4 supporting the hypothesis of SARS-CoV-2 susceptibility to TLRs like other Coronaviridae families. We also investigated whether the designed construct can provide immunity against the predominant delta and omicron variants. Thus, we tried to align the designed construct with the delta and omicron sequences to identify any mutation in structural regions of the virus. Among the twelve predicted and chosen epitopes in different areas of structural proteins of SARS-CoV-2 delta and omicron strains, only B-cell S^59-81^ epitope had mutations (A67V, 69del and 70 del). In this case, these mutations consist of a structural and/or charge mutation which may impact antibody binding. Since other chosen epitopes in the designed construct do not contain any mutation of SARS-CoV-2 delta and omicron strains, the designed construct can also provoke the immune system against delta and omicron strains. Moreover, our goal is further focused on T-cell epitopes as a therapeutic platform.

Up to now, various multiepitope constructs were designed against SARS-CoV-2 using immunoinformatics and *in silico* approaches [2, 40–42]. In 2020, a multiepitope subunit vaccine based on the spike protein (S) of SARS-CoV-2 was designed using *in silico* analysis [43]. In 2021, Yahaya *et al*. also designed a total of 454 amino acid sequences derived from CTL and HTL epitopes of SARS-CoV-2 structural proteins for vaccine development [2]. In another study, a SARS-coronavirus 2 genome-derived construct comprising 11 CD4, 12 CD8, 3 B cell, and 3 IFN-gamma epitopes along with an adjuvant β-defensin was designed to develop a multiepitope vaccine [42]. In the present study, we designed an effective multiepitope vaccine construct against SARS-COV-2. Different B-cell and T-cell epitopes were predicted and selected from three highly antigenic proteins of SARS-COV-2 (S, M & N). The selected epitopes significantly interacted with the HLA-binding alleles and showed 95.94% coverage of the world’s population. Vaccine construct included 229 amino acids by connecting 6 MHC class I and 4 MHC class II epitopes with suitable linkers. It was non-allergenic, antigenic, stable and flexible. Molecular docking demonstrated a stable and strong binding affinity of vaccine construct with human pathogenic toll-like receptors (TLRs). After design and synthesis of the multiepitope DNA sequence, its subcloning was performed in eukaryotic (pcDNA 3.1) and prokaryotic (pET) expression vectors (pcDNA-*s-n-m* and pET-*s-n-m*, respectively; S3 Fig). It should be mentioned that vaccine construct codons were optimized for subcloning into *Escherichia coli/* pET system for enhancing multiepitope peptide expression. *In vitro* delivery of pcDNA-*s-n-m* was performed to detect gene expression. Moreover, the S-N-M multiepitope peptide construct was generated in bacterial expression system.

Prompetchara *et al*. reported a construction strategy of DNA vaccine candidates expressing the full-length wild type SARS-CoV-2 spike (S) protein, S1 or S2 region (*i*.*e*., pCMVkan-S, S1 and–S2) and their immunogenicity in mice. All three DNA constructs induced high levels of specific binding IgG indicating a balance of IgG1/IgG2a response. The pCMVkan-S vaccine construct also induced the highest activity of T cells and IFN-γ secretion. Indeed, the full-length S antigen was more potent than the truncated spike (S1 or S2) in inducing of neutralizing antibody and strong T cell responses [44]. Moreover, two COVID-19 vaccines based on modified *vaccinia virus Ankara* (MVA) vectors expressing the entire SARS-CoV-2 spike (S) protein (MVA-CoV2-S) were developed and their immunogenicity was evaluated in mice using DNA/MVA or MVA/MVA prime/boost immunizations. Both vaccines induced potent polyfunctional S-specific CD4^+^ (mainly Th1) and CD8^+^ T-cell responses, with a T effector memory phenotype. However, DNA/MVA immunizations elicited higher T-cell immune responses. All vaccine regimens induced high titers of S-specific IgG antibodies. Two doses of this vaccine construct induced full inhibition of virus replication in the lungs [45]. Our study showed that three doses of the homologous S-N-M multiepitope peptide regimen could induce higher levels of total IgG and IgG1 than other groups. The higher ratios of IgG2a to IgG1 in groups immunized with the heterologous DNA + LL37 prime/ peptide boost (G2) and the homologous peptide prime/ peptide boost (G3) indicated direction of immune responses toward Th1 immune responses. Moreover, the homologous peptide prime/ peptide boost (G3) regimen induced high levels of IFN-gamma, TNF-alpha, IL-6, IL-21 and IL-15 cytokines, and Granzyme B.

Our *in vivo* finding of antibody induction differs from bioinformatic IgPred module we used. By using IgPred bioinformatic module we had a potential insight for keener observation in our *in vivo* study. However, this module which predicts the induction of different classes of antibodies based on sequence of the protein is not very accurate. Also, in the Raghava’s article of the IgPred accuracy, they implied that the learning machine technique with SVM-base models perform poor especially when positive and negative dataset is unbalanced [21]. This is also true for IFN-gamma prediction. All selected epitopes included in the designed construct were selected based on higher MHC binding rank which imply the affinity of binding epitopes to the MHC class I and II.

Leal *et al*. indicated that IL-6 regulates the phenotype of the immune response to a tuberculosis subunit vaccine. C57BL/6 mice immunized with this vaccine developed an increased level of interferon-γ secreted by CD4^+^ T cells directed toward strong Th1 response. This study showed that IL-6 plays a major role in the priming a Th1 response to a tuberculosis vaccine [46]. Espinosa-Ramos *et al*. developed an effective peptide vaccine against *H*. *pylori* targeting the conserved genes. Lymphoproliferation and spleen weights were significantly increased after immunization. Furthermore, a considerable increase in the level of IL-6 was observed in immunized and/ or infected animals. Peptide immunization protected 100% of mice against virulent *H*. *pylori* [47].

On the other hand, the immunogenicity of DNA-based vaccines was investigated in IL-15 KO mice (mice with the lack of IL-15 secretion). The results indicated that IL-15 is important for the generation of high number of antigen-specific CD8^+^T cells and the long-term memory CD8^+^T cells. Indeed, the granzyme B content was reduced in CD8^+^T cells of IL-15 KO mice compared to wild type mice [48, 49]. Another study showed that production of soluble IL-15Rα/IL-15 complexes (sIL-15 complexes) was increased in the serum of mice in response to IFN-α [50]. Moreover, the IL-15 immunotherapy may be a feasible strategy for COVID-19, as it induces innate immune responses via the induction of NK cells, CD8^+^ T cells, and T regulatory cells to neutralize Th2 cytokine storms, leading to decreased levels of IL-4, IL-5, and IL-13. These events could reduce SARS-CoV-2-induced inflammation and fibrosis through IFN-γ and IL-10, which suppress viral replication and decrease viral load [51].

IL-21 is induced by IL-6 in activated T cells, and effectively induces T_H_17 differentiation and suppresses Foxp3 expression. IL-21 is an autocrine cytokine necessary for T_H_17 differentiation, and used as a target for treating inflammatory diseases [52]. In this study, an increased level of IFN-gamma, TNF-alpha, IL-6, IL-21 and IL-15 was observed in groups immunized with S-N-M peptide + Montanide prime/ S-N-M peptide + Montanide boost (G3), and then pcDNA-*s-n-m* + LL37 prime/ S-N-M peptide + Montanide boost (G2). Higher levels of IL-5 and IL-10 were detected in group immunized with S-N-M peptide + Montanide prime/ S-N-M peptide + Montanide boost (G3). However, the ratio of IFN-gamma to IL-5 or IL-10 was high in group immunized with S-N-M peptide + Montanide prime/ S-N-M peptide + Montanide boost (G3), and then group immunized with pcDNA-*s-n-m* + LL37 prime/ S-N-M peptide + Montanide boost (G2). Indeed, immune response was directed toward Th1 response using these regimens. Regarding the increase of Th1-inducing cytokines, Granzyme B secretion (indicating *in vitro* CTL activity), and lymphocyte proliferation in groups immunized with S-N-M peptide + Montanide prime/ S-N-M peptide + Montanide boost (G3), and then pcDNA-*s-n-m* + LL37 prime/ S-N-M peptide + Montanide boost (G2), these regimens can be used to evaluate anti-SARS-CoV-2 effects in infected mice in future. Moreover, the use of other adjuvants and/ or other routes of vaccination (*e*.*g*., intramuscular or nasal administration) can be considered for the best regimens in the next studies.

## Conclusion

As mentioned in our previous study, the *in silico* data require validation by *in vivo* experiments. Thus, in this study, we tried to validate our designed multiepitope construct. In conclusion, we used immunoinformatics analysis on the S, M and N proteins of SARS-CoV-2 for development of a safe and potent multiepitope vaccine candidate. This vaccine construct against three major structural proteins of SARS-CoV-2 was designed based on robust vaccine design criteria including non-allergenicity, 3D prediction, refinement and validation, discontinuous B-cell epitope prediction, docking and effectiveness of molecular interaction with their respective human leukocyte antigen (HLA) alleles and TLRs and the ability to induce immunity against variants of concern such as delta and omicron variants. The high-ranked epitopes of B- and T-cells of S, N and M proteins were connected to each other using the flexible KK, GPGPG and AAY proteolytic linkers. Molecular docking analysis showed a positive association of this construct with Toll-like receptor 3. These *in silico* data required validation by *in vitro* and *in vivo* experiments. Our data indicated that the multiepitope S-N-M peptide construct combined with Montanide 720 in homologous regimen could significantly stimulate total IgG, IgG1, IgG2a, IFN-γ, TNF-α, IL-15, IL-21, IL-6, IL-5 and IL-10, and Granzyme B secretion at three weeks and three months after the last injection. It was important that the levels of antibodies, cytokines and granzyme B were almost stable in three months after the last injection. Moreover, LL37 cell penetrating peptide could effectively increase the potency of DNA construct in enhancing immune responses as compared to the naked DNA. Indeed, LL37 peptide delivers low doses of DNA (~ 5 μg) as compared to the naked DNA (50 μg) with higher efficiency in inducing immune responses.

## Supporting information

S1 Fig The synthesized polyepitope DNA construct with the restriction enzymes (*Bam*HI: GGATCC: Brown color; *Xho*I: CTCGAG: Red color; *Hind*III: AAGCTT: Blue color; *Not*I: GCGGCCGC: Orange color), and His-tag (CACCACCACCACCACCAC: Green color). (TIF)

S2 Fig Confirmation of the recombinant pcDNA-*s-n-m* (A), and pET24a-*s-n-m* (B) by double digestion using restriction enzymes (Lane 1). MW is a molecular size marker (1 kb, Smobio).
(TIF)

S3 Fig A schematic model of study design. (TIF)

S1 Raw images The original unadjusted, uncropped blot/gel images for Figs 5 and 6, and S2 Fig. (RAR)

S1 Table Prediction of linear B-cell epitopes in the designed S-N-M multiepitope peptide. (TIF)
