# Supplementary figures and images for: Immunological investigation of a multiepitope peptide vaccine candidate based on main proteins of SARS-CoV-2 pathogen
Source: PLoS One. 2022 Jun 9;17(6):e0268251. doi: 10.1371/journal.pone.0268251 (PMC9182696; doi:10.1371/journal.pone.0268251)

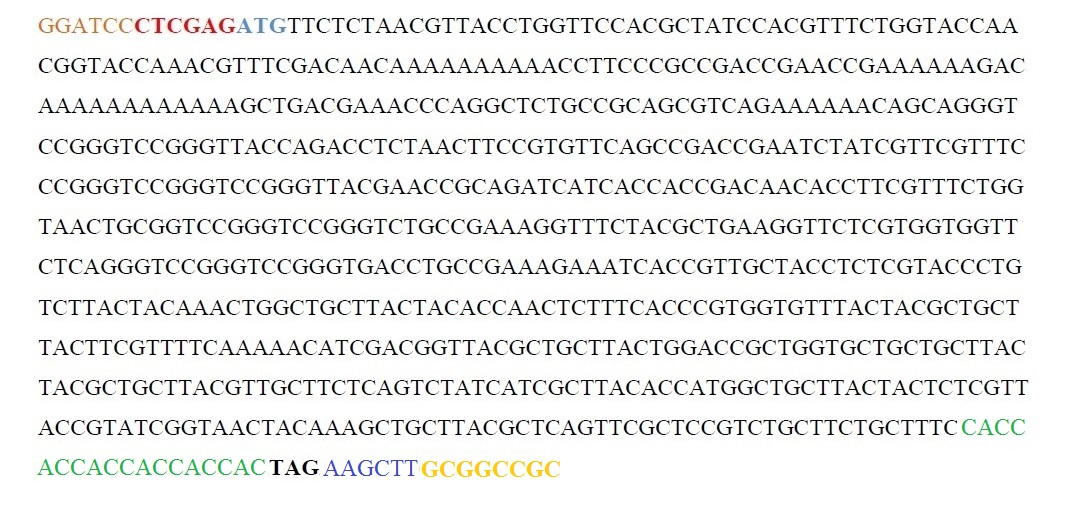

Supplement: S1 Fig — (TIF) [file pone.0268251.s001.tif]

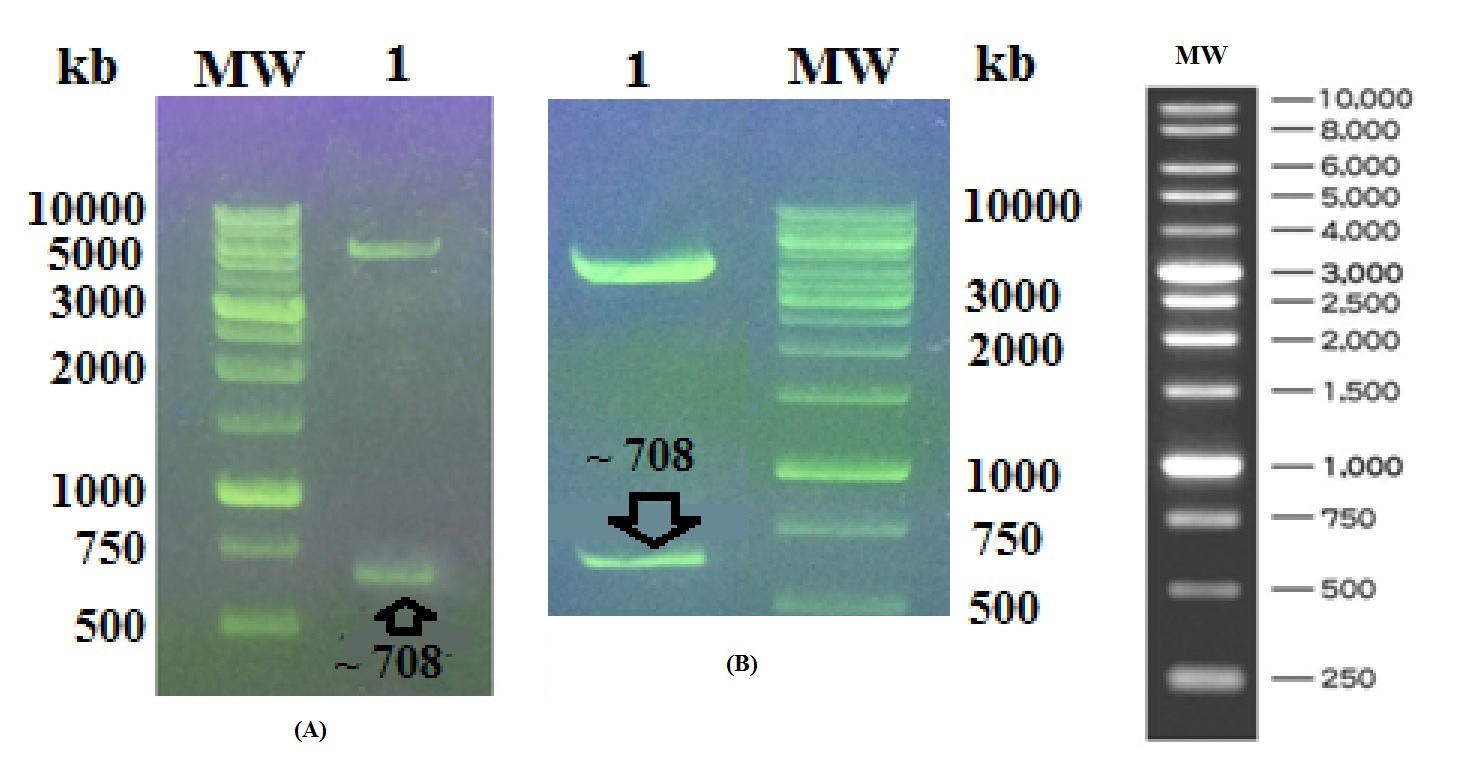

Supplement: S2 Fig — MW is a molecular size marker (1 kb, Smobio). (TIF) [file pone.0268251.s002.tif]

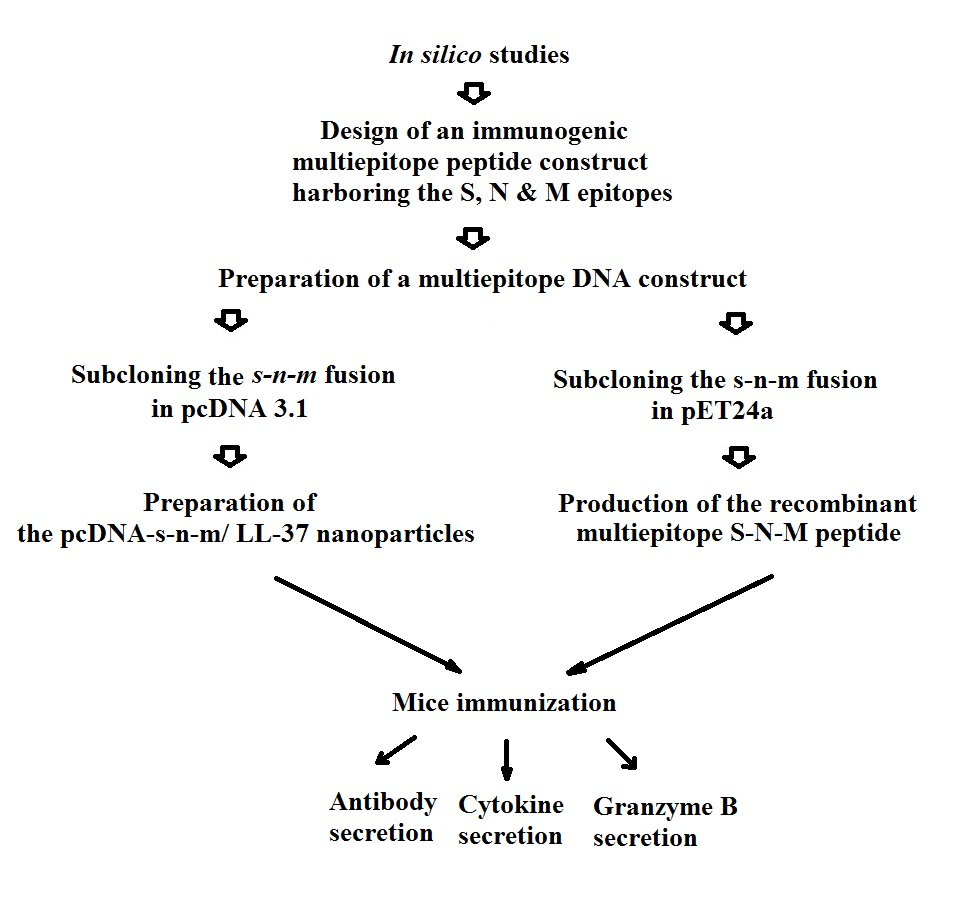

Supplement: S3 Fig — (TIF) [file pone.0268251.s003.tif]

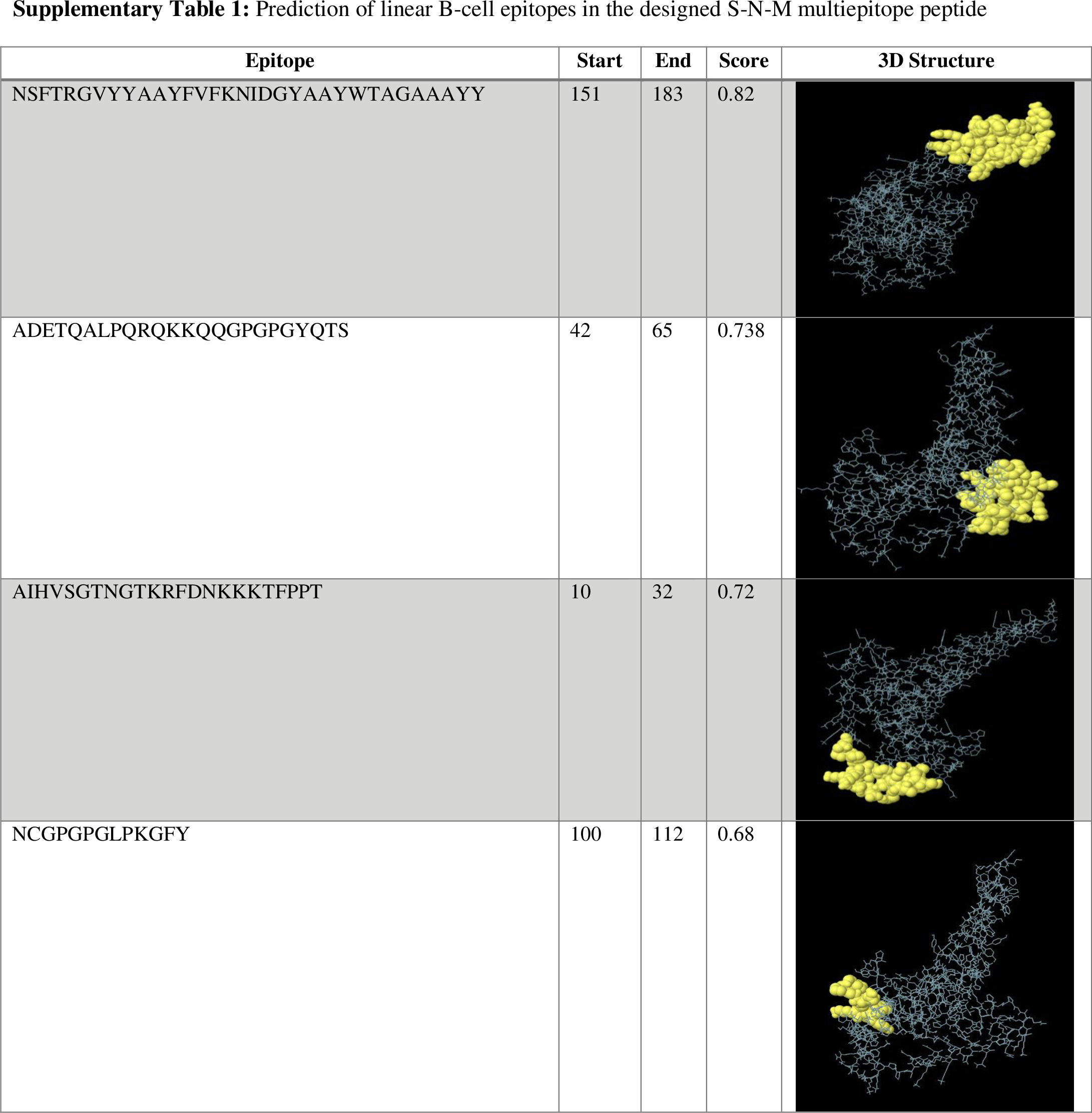

Supplement: S1 Table — (TIF) [file pone.0268251.s005.tif]
